# Genetically Encoded Protein Phosphorylation in Mammalian Cells

**DOI:** 10.1016/j.chembiol.2018.05.013

**Published:** 2018-09-20

**Authors:** Václav Beránek, Christopher D. Reinkemeier, Michael S. Zhang, Alexandria D. Liang, Gene Kym, Jason W. Chin

**Affiliations:** 1 Medical Research Council Laboratory of Molecular Biology, Francis Crick Avenue, Cambridge, UK

**Keywords:** genetic code expansion, phosphorylation, phosphoserine, mammalian cells, phosphoserine analog, synthetic biology

## Abstract

Protein phosphorylation regulates diverse processes in eukaryotic cells. Strategies for installing site-specific phosphorylation in target proteins in eukaryotic cells, through routes that are orthogonal to enzymatic post-translational modification, would provide a powerful route for defining the consequences of particular phosphorylations. Here we show that the SepRS^v1.0^/tRNA^v1.0^_CUA_ pair (created from the *Methanococcus maripaludis* phosphoseryl-transfer RNA synthetase [*Mm*SepRS]/*Methanococcus janaschii* [*Mj*]tRNA_GCA_^Cys^ pair) is orthogonal in mammalian cells. We create a eukaryotic elongation factor 1 alpha (EF-1α) variant, EF-1α-Sep, that enhances phosphoserine incorporation, and combine this with a mutant of eRF1, and manipulations of the cell’s phosphoserine biosynthetic pathway, to enable the genetically encoded incorporation of phosphoserine and its non-hydrolyzable phosphonate analog. Using this approach we demonstrate synthetic activation of a protein kinase in mammalian cells.

## Highlights

• SepRS^v1.0^/tRNA^v1.0^_CUA_ is an orthogonal pair in mammalian cells
• Phosphoserine is genetically directed into proteins in mammalian cells
• Phosphonate analog of phosphoserine is stably incorporated in mammalian cells
• Encoded phosphonate analog enables synthetic kinase activation

## Graphical Abstract

**Figure:**
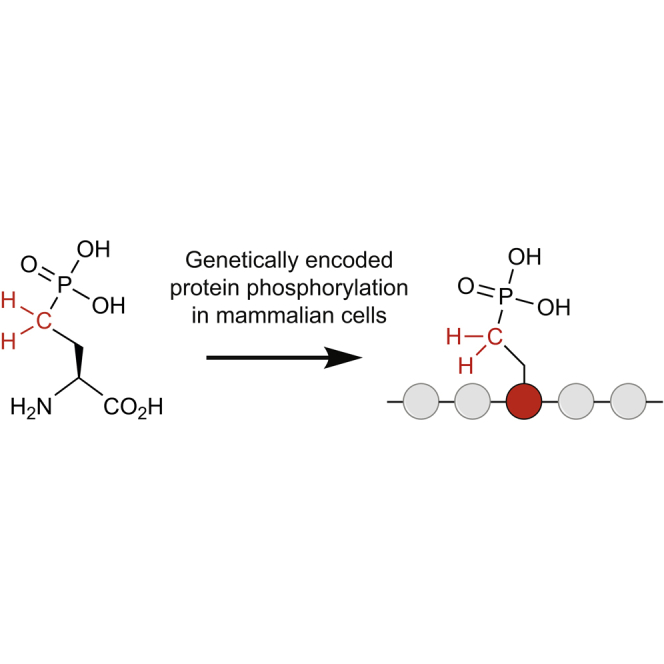

## Teaser

Beránek et al. describe an aminoacyl-tRNA synthetase/tRNA pair that is orthogonal in mammalian cells. They demonstrate that this pair can be used to incorporate phosphoserine and a stable phosphonate analog. By encoding the phosphonate analog in a kinase activation loop, they synthetically activate the kinase.

## Introduction

Protein phosphorylation is a key post-translational modification that expands the complexity of protein function, and regulates diverse biological processes in eukaryotic systems (Manning et al., 2002). Thousands of phosphorylation sites have been discovered (Olsen et al., 2006), but the consequences of protein phosphorylation can be hard to determine, as manipulation of the kinases that install phosphorylation commonly have pleiotropic effects, and so-called phosphomimetic mutations to aspartic acid and glutamic acid are chemically distinct (Mandell et al., 2007) and often fail to recapitulate the molecular and phenotypic consequences of phosphorylation (Roberts-Galbraith et al., 2010).

Genetic code expansion, using orthogonal aminoacyl-tRNA synthetase/tRNA_CUA_ pairs, has enabled the site-specific incorporation of diverse non-canonical amino acids (ncAAs) into proteins in cells and animals (Chin, 2014). One key application of this approach is in understanding the role of post-translational modifications in regulating the biological functions of proteins. Directly genetically encoding ncAAs, corresponding to post-translationally modified versions of natural amino acids, into proteins has enabled the synthesis of recombinant proteins bearing defined post-translational modifications, and led to numerous insights into how these modifications regulate protein structure and function (Davis and Chin, 2012, Chin, 2017).

Recently, a phosphoseryl-tRNA synthetase (SepRS)/tRNA_GCA_ pair that directs the first step in a tRNA-dependent cysteine biosynthesis pathway in certain methanogens (Sauerwald et al., 2005) has been co-opted for the site-specific incorporation of phosphoserine (**1**) into recombinant proteins in *Escherichia coli* (Park et al., 2011, Rogerson et al., 2015). Since SepRS recognizes the anticodon of tRNA_GCA_, the SepRS/tRNA_CUA_ pair, in which the GCA anticodon was simply substituted by CUA, was a very inefficient amber suppressor. In recent work, we demonstrated that phosphoserine could be efficiently incorporated into proteins in *E. coli* using an evolved SepRS/tRNA_CUA_ pair (Rogerson et al., 2015). This pair, in which SepRS and the anticodon stem and anticodon loop of tRNA_CUA_ were evolved to function efficiently, referred to herein as the SepRS^v1.0^/tRNA^v1.0^_CUA_ pair, has been used to produce a number of site-specifically phosphorylated proteins for structural and functional studies (Rogerson et al., 2015, Huguenin-Dezot et al., 2016, Burgess et al., 2018, Dickson et al., 2018). We also demonstrated that by manipulating phosphoserine biosynthesis in *E. coli*, a non-hydrolyzable, phosphonate analog of phosphoserine (**2**) could be incorporated into proteins using the SepRS^v1.0^/tRNA^v1.0^_CUA_ pair (Rogerson et al., 2015), and that the pair can be further evolved to incorporate phosphothreonine into proteins in *E. coli* (Zhang et al., 2017).

The ability to encode phosphoserine, and its non-hydrolyzable analogs, into defined sites in proteins in mammalian cells would facilitate an understanding of the molecular and cellular consequences of this modification. Unlike approaches that manipulate kinases and phosphatases, that have many targets in the cell, orthogonal routes to installing site-specific phosphorylation may directly address the consequences of modifying a particular site on a particular protein.

Orthogonal routes to installing other post-translational modifications have begun to emerge. We recently explored the genetic encoding of acetyl-lysine into chromatin (Elsässer et al., 2016), and complementary work explored directing protein ubiquitination into chromatin via protein *trans*-splicing (David et al., 2015); both these approaches modify only a small fraction of histones, but have led to important conclusions about the consequences of these modifications for gene expression and cellular interactions.

Protein phosphorylation is commonly activating and phosphoproteomics studies reveal that proteins are naturally regulated by sub-stoichiometric phosphorylation, with occupancies of less than 30% reported on the majority of phosphorylation sites (Olsen et al., 2010). Therefore, routes to the sub-stoichiometric installation of phosphorylated amino acids into proteins in mammalian cells will be useful.

Here we demonstrate that the SepRS^v1.0^/tRNA^v1.0^_CUA_ pair is orthogonal in mammalian cells and can be used, in combination with other engineered translational components, to site-specifically and co-translationally incorporate phosphoserine and its non-hydrolyzable phosphonate analog into proteins expressed in mammalian cells. Moreover, we demonstrate the synthetic activation of a protein kinase in mammalian cells by encoding a phosphonate analog at a site that is normally phosphorylated by an upstream kinase.

## Results

### Expressing SepRS^v1.0^/tRNA^v1.0^_CUA_ in Human Cells

**Figure 1:**
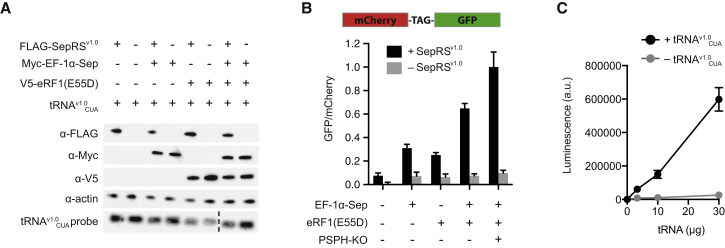
The SepRS^v1.0^/tRNA^v1.0^_CUA_ Pair Enables pSer Incorporation in Mammalian Cells (A) Expression of FLAG-SepRS^v1.0^, Myc-EF-1α-Sep, V5-eRF1(E55D) and tRNA^v1.0^_CUA_ in HEK293 cells detected via western and northern blots. Dashed line indicates removal of irrelevant lanes. (B) SepRS^v1.0^/tRNA^v1.0^_CUA_ pair directs pSer incorporation in mammalian cells, co-expression of eEF1-αSep, eRF1(E55D), and knockout of PSPH gene increase pSer incorporation. Readthrough of UAG codon is determined by ratio of GFP to mCherry fluorescence from the mCherry-TAG-GFP reporter. Data represent mean ± SEM for at least three biological replicates. (C) Recombinant SepRS^v1.0^ is incubated with total tRNA extracted from control mammalian cells (– tRNA^v1.0^_CUA_) or cells expressing tRNA^v1.0^_CUA_ (+tRNA^v1.0^_CUA_). The aminoacylation in each reaction is determined by measuring the AMP production with the AMP-Glo assay. Data represent mean ± SEM for triplicate reactions.

We first demonstrated that the SepRS^v1.0^/tRNA^v1.0^_CUA_ pair can be expressed in mammalian cells. We were able to detect FLAG-tagged SepRS^v1.0^, following transfection of HEK293 cells with the corresponding gene, by western blot (Figure 1A). Similarly, upon expressing four repeats of the gene encoding tRNA^v1.0^_CUA_ from extragenic U6 promoters, we detected expression of tRNA^v1.0^_CUA_ by northern blot (Figure 1A). These experiments demonstrate that SepRS and tRNA^v1.0^_CUA_ can be expressed in human cells.

To assess the functionality of the SepRS^v1.0^/tRNA^v1.0^_CUA_ pair in human cells, we measured the readthrough of the amber stop codon in an mCherry-TAG-GFP construct via the ratio of GFP and mCherry fluorescence. Expression of tRNA^v1.0^_CUA_ alone led to minimal readthrough of the amber stop codon, consistent with this tRNA being minimally aminoacylated by endogenous synthetases. This observation suggests that tRNA^v1.0^_CUA_ is orthogonal in mammalian cells (Figure 1B). Addition of SepRS^v1.0^ led to an increase in amber suppression consistent with the SepRS^v1.0^-mediated aminoacylation of tRNA^v1.0^_CUA_ with phosphoserine, which is present in mammalian cells as a biosynthetic precursor to serine (Ichihara and Greenberg, 1957).

In *E. coli*, the incorporation of phosphoserine into proteins by the SepRS^v1.0^/tRNA^v1.0^_CUA_ pair can be enhanced by a mutant form of EF-Tu, named EF-Sep (Park et al., 2011), which may accommodate the phosphorylated amino acid. EF-1α is the eukaryotic equivalent of EF-Tu, and delivers aminoacylated tRNAs to the eukaryotic ribosome (Carvalho et al., 1984). We therefore asked whether SepRS^v1.0^-dependent readthrough of the amber stop codon in mammalian cells can be enhanced by a mutant of EF-1α. To design a mutant of EF-1α for pSer incorporation in mammalian cells, we aligned the EF-Tu and EF-1α sequences and identified the amino acids in EF-Tu that were mutated to create EF-Sep (Figures S1A and S1B). We then created the corresponding mutations (L77R, Q251N, D252G, V264S, N307W) in EF-1α to create EF-1α-Sep, and demonstrated that this mutant was expressed in mammalian cells (Figure 1A). Co-expression of EF-1α-Sep with the SepRS^v1.0^/tRNA^v1.0^_CUA_ pair led to an increase in the readthrough of the amber stop codon in mCherry-TAG-GFP (Figure 1B).

We have previously shown that the efficiency of ncAA incorporation, using the PylRS/tRNA_CUA_ pair, in mammalian cells can be enhanced by expressing an eRF1(E55D) mutant of eRF1 (Schmied et al., 2014, Kolosov et al., 2005); the eukaryotic release factor that terminates protein synthesis at all three stop codons. eRF1(E55D) does not efficiently terminate protein synthesis in response to amber codons, and its expression minimizes release factor competition with amber suppressor tRNAs at the amber stop codon, while maintaining termination on the other two stop codons. Co-expression of eRF1(E55D) with the SepRS^v1.0^/tRNA^v1.0^_CUA_ pair was confirmed by immunoblotting (Figure 1A) and led to a substantial increase in readthrough of the amber stop codon (Figure 1B).

Next, we combined the SepRS^v1.0^/tRNA^v1.0^_CUA_ with both EF-1α-Sep and eRF1(E55D). This combination led to the greatest readthrough of the amber codon in the presence of SepRS^v1.0^ (Figure 1B). These data are consistent with a model in which SepRS^v1.0^, which is known to selectively recognize phosphoserine (Hauenstein et al., 2008), aminoacylates the orthogonal tRNA^v1.0^_CUA_ with phosphoserine in mammalian cells, creating pSer-tRNA^v1.0^_CUA_. pSer-tRNA^v1.0^_CUA_ is taken to the ribosome with the aid of EF-1α-Sep, where it is more efficiently decoded in response to the amber codon in the presence of eRF1(E55D). All further experiments were carried out using the SepRS^v1.0^/tRNA^v1.0^_CUA_ pair with EF-1α-Sep and eRF1(E55D). To verify that transfection of the SepRS^v1.0^/tRNA^v1.0^_CUA_, EF-1α-Sep, and eRF1(E55D) does not compromise cellular fitness, we compared the cell viability with cells transfected with control plasmid (Figure S1C) and saw no significant difference. We note that both the altered translation factors (EF-1α-Sep and eRF1[E55D]) mediate some SepRS^v1.0^/tRNA^v1.0^_CUA_ pair independent readthrough of the amber stop codon; this may result from near-cognate decoding of the amber codon by endogenous tRNAs (Roy et al., 2015).

### Phosphoserine Phosphatase Deletion Increases Intracellular pSer

Does the intracellular concentration of phosphoserine limit the efficiency of its incorporation using our system in mammalian cells? To address this question we extracted metabolites from cells and found that the phosphoserine levels were less than 100 μM *in vivo* (Figures S1D and S1E). Since the *Km* of SepRS for phosphoserine is approximately 270 μM (Hauenstein et al., 2008), we reasoned that increasing the pSer concentration in cells might increase the efficiency of its incorporation into proteins. In mammals, phosphoserine phosphatase (PSPH) converts phosphoserine to serine in the last step of serine biosynthesis (Snell, 1984) and we hypothesized that knocking out PSPH might lead to an increase in intracellular phosphoserine levels and allow us to test the effect of phosphoserine levels on SepRS^v1.0^-mediated incorporation into proteins. We performed CRISPR-Cas9-mediated knockout of PSPH in HEK293, and confirmed the knockout by genotyping and western blot (Figures S1F and S1G). In the resulting cell line, HEK293/PSPH-KO, the intracellular pSer concentration increased by at least 400 ± 60 μM (SD) over HEK293 (Figure S1H). This increase in intracellular phosphoserine led to a measurable increase in phosphoserine incorporation in response to the amber codon in the HEK293/PSPH-KO (Figure 1B). We conclude that phosphoserine incorporation levels in mammalian cells can be increased by PSPH deletion. Overall, the use of EF-1α-Sep, eRF1(E55D), and the PSPH knockout increase the efficiency of SepRS^v1.0^/tRNA^v1.0^_CUA_-mediated amber suppression by more than an order of magnitude.

### SepRS^v1.0^ Is Orthogonal with Respect to Mammalian tRNA

Next we demonstrated that SepRS^v1.0^ is selective for tRNA^v1.0^_CUA_ with respect to the mammalian tRNAs. We isolated total tRNA from HEK293 cells (−tRNA^v1.0^_CUA_) and from HEK293 cells expressing tRNA^v1.0^_CUA_ (+tRNA^v1.0^_CUA_), in which tRNA^v1.0^_CUA_ makes up less than 10% of the total mammalian tRNA pool (Figure S1I). We subjected each tRNA pool to *in vitro* aminoacylation with phosphoserine using purified SepRS^v1.0^. We followed the extent of aminoacylation as a function of total tRNA concentration by measuring AMP production (Mondal et al., 2017). For +tRNA^v1.0^_CUA_ we observed an increase in aminoacylation with total tRNA concentration, while for −tRNA^v1.0^_CUA_ we observed minimal aminoacylation at all tRNA concentrations tested (Figure 1C). Our results demonstrate that SepRS^v1.0^ does not substantially aminoacylate endogenous mammalian tRNAs but selectively aminoacylates tRNA^v1.0^_CUA_. We conclude that SepRS^v1.0^ is orthogonal with respect to the tRNAs in mammalian cells.

### Encoded pSer Is Post-translationally Converted to Ser

**Figure 2:**
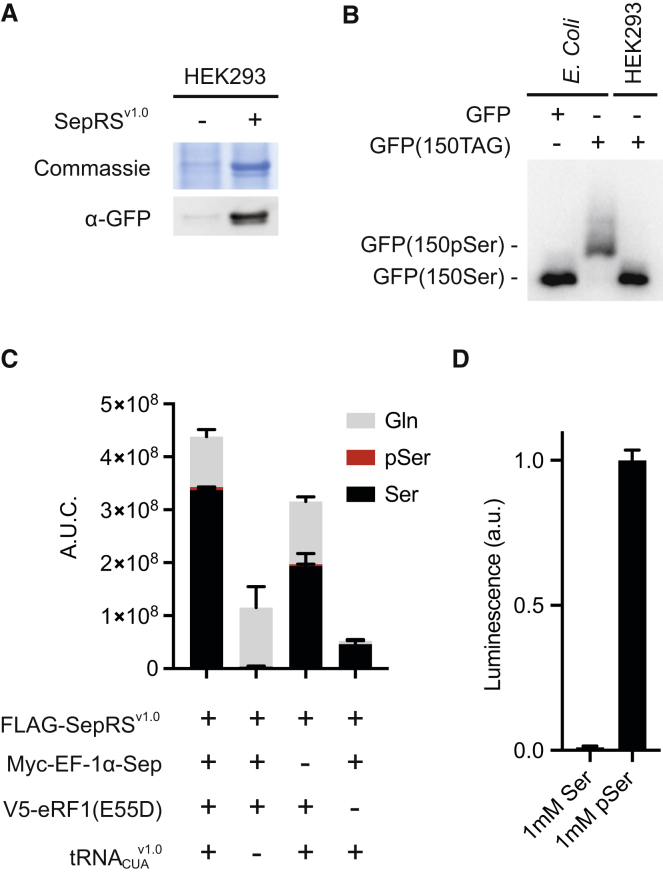
SepRS^v1.0^/tRNA^v1.0^_CUA_ Directs pSer into Proteins, Where pSer Is Post-Translationally Dephosphorylated (A) Coomassie-stained SDS-PAGE gel and western blot of purified GFP from HEK293 cells. (B) pSer is not maintained post-translationally in GFP expressed in mammalian cells. The Phos-tag SDS-PAGE gel leads to a mobility shift in phosphorylated proteins via chelation of the phosphate in the gel. GFP and GFP(150pSer) standards were produced in *E. coli* as described previously (Rogerson et al., 2015), and define the mobility of phosphorylated and non-phosphorylated GFP. GFP was detected by immunoblotting. (C) A.U.C. is the area under the curve of the extracted ion chromatograms for peptide LEYNFNSH[X]VYITADK in MS1 of the tryptic LC-MS/MS. Identity of the peptides was confirmed by MS/MS analysis (see Figure S2). Data represent means ± SD for two biological replicates. (D) SepRS^v1.0^ is selective for phosphoserine over serine. Recombinant SepRS^v1.0^ was incubated with total tRNA extracted from mammalian cells expressing tRNA^v1.0^_CUA_. The aminoacylation in each reaction was determined by measuring the AMP production with AMP-Glo assay. Data represent mean ± SEM for reactions in four replicates.

To investigate the identity of the amino acid incorporated into proteins in response to the amber codon we created a streamlined expression system in which SepRS, eRF1(E55D), EF-1α-Sep and four copies of tRNA^v1.0^_CUA_ are combined on a single plasmid. Co-transfection of this plasmid with a plasmid containing GFP(150TAG) and four copies of tRNA^v1.0^_CUA_ into HEK293 cells enabled expression and purification of the resulting GFP (Figure 2A).

To investigate whether phosphoserine was present in GFP expressed in mammalian cells, we prepared authentic standards of GFP bearing phosphoserine at position 150 (GFP[150pSer]), using a previously described *E. coli* expression system (Rogerson et al., 2015), and GFP bearing serine at position 150 (GFP[150Ser]). We analyzed the samples on phos-tag-SDS-PAGE, a well-established technique in which phosphorylated proteins are resolved from non-phosphorylated proteins and the extent of phosphorylation can be quantified (Kinoshita et al., 2006, Kinoshita et al., 2009).

As expected, the mobility of GFP(150pSer) was retarded with respect to that of GFP(150Ser) (Figure 2B). The majority of GFP expressed from GFP(150TAG) in mammalian cells migrated with the GFP150Ser standard prepared in *E. coli*, and we were not able to detect any phosphorylated GFP. This result demonstrates that the majority of the amino acid at position 150 in GFP, expressed from GFP(150TAG) in mammalian cells, is not phosphorylated.

To investigate the identity of amino acids incorporated in response to the amber codon in GFP, we performed electrospray ionization tandem mass spectrometry (ESI-MS/MS) on tryptic digests of GFP expressed in mammalian cells. The extracted ion chromatographs of peptides at the MS1 level reveal that serine, glutamine, and traces of phosphoserine are found in GFP produced from GFP(150TAG) in mammalian cells containing SepRS^v1.0^/tRNA^v1.0^_CUA_, EF-1α-Sep, and eRF1(E55D) (Figure 2C). Control experiments show that the relevant peptides containing pSer and Ser ionize with comparable efficiency (Figures S2A and S2B).

We suspected that the glutamine incorporation we observe in response to the amber codon in mammalian cells may result from suppressor tRNA independent incorporation of glutamine upon expressing eRF1(E55D) in mammalian cells (Roy et al., 2015), consistent with the SepRS^v1.0^ independent readthrough of the amber codon observed with eRF1(E55D) in our initial system (Figure 1B). Protein expression in the absence of eRF1(E55D) led to a decrease in GFP synthesis (Figure 2C), as expected based on the role of this mutant in decreasing the efficiency of translational termination at the amber stop codon. It also led to a substantial increase in the ratio of serine to glutamine at position 150 of GFP (Figure 2C), consistent with a decrease in non-cognate decoding of the TAG codon when the normal termination machinery is active. In contrast, protein expression in the absence of EF-Sep led to a decrease in protein, and an increase in the ratio of glutamine to serine at position 150 (Figure 2C), consistent with this factor increasing the delivery of phosphoseryl-tRNA^v1.0^_CUA_ to the ribosome and post-translational dephosphorylation of phosphoserine. Finally, protein expression in the absence of the amber suppression machinery (−tRNA^v1.0^_CUA_) but in the presence of eRF1(E55D) led to a decrease in GFP, and the protein predominantly incorporated glutamine at position 150 (Figure 2C), indicating that the glutamine incorporation is not mediated by the SepRS^v1.0^/tRNA^v1.0^_CUA_ pair. These observations are consistent with previous reports of glutamine incorporation in response to premature termination codons in eukaryotic mRNAs (Roy et al., 2015).

The observed serine incorporation could formally result from either the co-translational incorporation of serine by SepRS^v1.0^/tRNA^v1.0^_CUA_ or the incorporation of phosphoserine followed by its post-translational dephosphorylation by phosphatases in cells. SepRS is known to be highly selective for phosphoserine over serine. Previous work has shown that SepRS selectively forms the aminoacyl-adenylate of phosphoserine over that of serine by a factor of 10^4^ in the first step of aminoacylation (Hauenstein et al., 2008), and the overall kinetics of aminoacylation of serine with this system were too low to measure; these observations suggest that SepRS discriminates against serine by much more than 10^4^-fold in the overall aminoacylation reaction (Hauenstein et al., 2008). Consistent with these prior observations, the aminoacylation of tRNA^v1.0^_CUA_ with 1 mM serine by SepRS^v1.0^ was negligible (Figure 2D). In contrast, tRNA^v1.0^_CUA_ was efficiently aminoacylated by SepRS^v1.0^ in the presence of 1 mM phosphoserine. These observations further confirm that tRNA^v1.0^_CUA_ is not aminoacylated with serine by SepRS^v1.0^.

Our observations suggest that in mammalian cells, our system directs the co-translational incorporation of phosphoserine in response to the amber codon, and that most of the genetically encoded phosphorylation in GFP is post-translationally dephosphorylated to serine. As numerous phosphatases for serine and threonine residues are present in mammalian cells (Hornbeck et al., 2004), this observation is not surprising.

### Encoding a Non-hydrolyzable pSer

**Figure 3:**
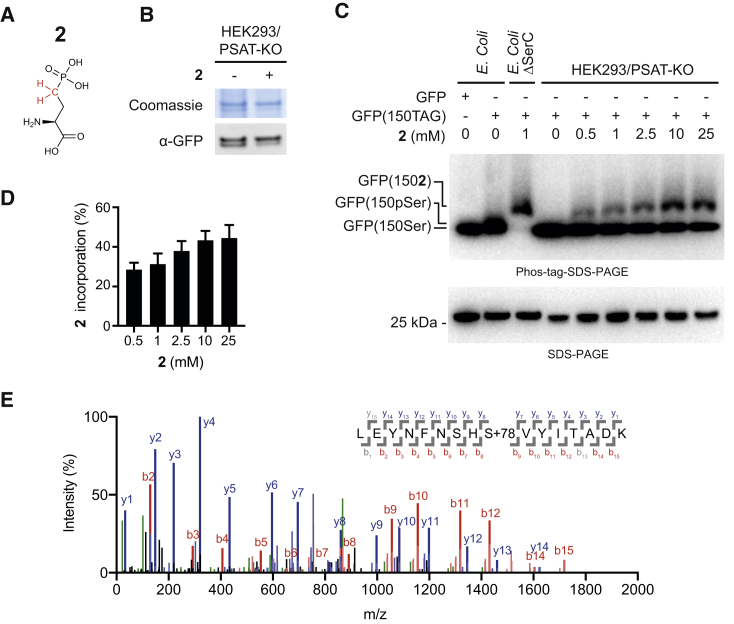
Encoding Non-Hydrolyzable Phosphonate Analogue (2) of pSer in Genetically Engineered Mammalian Cells (A) Phosphonate analogue of phosphoserine (**2**) used in this study. (B) Protein expression in the PSAT-KO. Coomassie-stained SDS-PAGE gel and western blot of purified GFP from HEK293/PSAT-KO overexpressing PSPH. Amino acid **2** was used at 10 mM. (C) Separation on phos-tag SDS-PAGE gel followed by immunoblotting is consistent with incorporation of **2** in the HEK293/PSAT-KO cell line overexpressing PSPH. GFP, GFP(150pSer) and GFP(150[**2**]) standards were produced in *E. coli* as described previously (Rogerson et al., 2015). (D) Quantification of the relative incorporation of **2** as a result of increasing concentration of **2** added to the cells. Data represent mean ± SEM for three biological replicates. (E) Incorporation of **2** into GFP(150TAG) reporter at the genetically encoded site was verified by ESI-MS/MS. See also Figure S3.

Next we asked whether we could increase the fraction of phosphorylated protein by encoding a non-hydrolyzable phosphonate analog of pSer (**2**) (Figure 3A) in cells depleted in phosphoserine and fed **2**. We first established that **2** can enter mammalian cells and is a good substrate for SepRS^v1.0^. Supplementing the growth media with 2 mM and 10 mM **2** led to intracellular concentrations of 3.64 ± 0.39 mM and 12.47 ± 0.80 mM, respectively (Figure S3A), demonstrating that we can generate millimolar concentrations of amino acid **2** in the cytosol. *In vitro* aminoacylation experiments confirm that SepRS^v1.0^ can efficiently aminoacylate tRNA^v1.0^_CUA_ with **2** (Figure S3B), in line with our previous observations incorporating the amino acid in *E. coli* (Rogerson et al., 2015).

Since pSer is biosynthesized from phosphohydroxypyruvate via the action of PSAT and converted to serine via the action of PSPH (Ichihara and Greenberg, 1957), we reasoned that phosphoserine levels resulting from this pathway could be minimized by both the deletion of PSAT and the overexpression of PSPH. We created a CRISPR-Cas9-mediated knockout cell line, HEK293/PSAT-KO, and confirmed this knockout by genotyping and western blot (Figures S3C and S3D). pSer levels were already below the detection limit of our liquid chromatography-mass spectrometry (LC-MS) assay in HEK293 cell metabolite extractions, and we could not measure these levels in the HEK293/PSAT-KO strain. We found that the effect of the PSAT knockout and PSPH overexpression on serine levels was modest, reducing them from 362 ± 52 μM in HEK293 cells to 201 ± 22 μM and 215 ± 14 μM (all errors are SD) for the PSAT knockout and PSAT knockout with PSPH overexpression, respectively (Figure S3E).

We co-transfected HEK293/PSAT-KO cells with two plasmids: one encoding SepRS^v1.0^, EF-1α-Sep, eRF1(E55D), PSPH, and four copies of tRNA^v1.0^_CUA_, and one encoding GFP(150TAG) and four copies of tRNA^v1.0^_CUA_. GFP was expressed from these cells via readthrough of the amber codon. Protein production in these cells was comparable in the presence and absence of **2** (Figure 3B).

To investigate whether our system enables **2** to compete with phosphoserine for SepRS^v1.0^-mediated incorporation into proteins in mammalian cells, we took advantage of our ability to prepare proteins that site-specifically incorporate either phosphoserine or **2** in *E. coli* (Rogerson et al., 2015). We produced authentic standards of GFP incorporating pSer at position 150 (GFP[150pSer]) and GFP incorporating **2** at position 150 (GFP[150**2**]) in *E. coli* (Figures S2A–S2C), and subjected these (along with GFP encoding serine at position 150 [GFP(150Ser)]) to phos-tag-SDS-PAGE (Figure 3C).

We discovered that the phosphonate in **2** leads to a dramatic decrease in mobility of GFP(150**2**), with respect to both GFP and GFP(150pSer), in phos-tag-SDS-PAGE (we ran these gels under conditions where GFP150Ser and GFP150pSer are not resolved, as longer runs led to diffuse bands of GFP150**2**). This result demonstrates that GFP incorporating **2** can be resolved by its mobility in phos-tag-SDS-PAGE (Figure 3C). GFP expressed from GFP(150TAG) in mammalian cells deleted for PSAT, and expressing PSPH, SepRS^v1.0^, EF-1α-Sep, eRF1(E55D), and tRNA^v1.0^_CUA_, and provided with **2** led to two bands on phos-tag-SDS-PAGE; one band co-migrated with the authentic standard for GFP(150**2**) and the other with the authentic standards for GFP(150Ser) and GFP150pSer (Figure 3C, top panel). Standard SDS-PAGE analysis of the same sample produced a single band for each sample, confirming that the slower migration of the upper band in phos-tag SDS-PAGE is due to a different interaction of **2** with the phos-tag gel matrix (Figure 3C). The GFP(150**2**) fraction of the total GFP produced increased with the concentration of **2** in the growth media and reached 49% ± 11% (SEM) for 25 mM **2**. MS (Figures 3E and S3F–S3I) revealed that the GFP produced contained either glutamine or **2**; this suggests that **2** can outcompete pSer at the active site of SepRS (control experiments demonstrate that the relevant peptides containing pSer and **2** ionize comparably) (Figures S2A–S2C), but that the amber suppressor tRNA does not entirely outcompete glutamine incorporation in the presence of eRF1(E55D). We note that the ratio of **2** to glutamine in these experiments is lower than the ratio of serine to glutamine when phophoserine is incorporated (Figure 2C); this may be a result of the different genetic backgrounds used for the two experiments.

Phosphorylation is often an activating, dominant modification and glutamine incorporation in place of serine is very unlikely to have phenotypic consequences (and these can be explicitly ruled out by testing the phenotype of an Ser to Gln mutation). Moreover, proteomics studies suggest that the majority of proteins are naturally regulated by sub-stoichiometric phosphorylation (<30%) (Olsen et al., 2010). Thus, the sub-stoichiometric genetic encoding of phosphorylated amino acids in mammalian cells should not substantively limit the utility of our system for studies in mammalian cells.

### Kinase Activation by Genetically Encoded Phosphorylation

Next we explicitly demonstrated that genetically encoding **2** at a biologically relevant site of serine phosphorylation enables synthetic activation of a protein kinase (MEK1) in mammalian cells. It is well established that phosphorylation at serine 218 and serine 222 of MEK1, upon stimulation of upstream kinases by growth factors, leads to activation of MEK1 and phosphorylation of its substrates, notably ERK1/2 (Shaul and Seger, 2007). Serine to aspartic acid substitutions at positions 218 and 222 can activate MEK1 (Huang and Erikson, 1994). MEK1 is also phosphorylated at several other sites (Hornbeck et al., 2004) and the native protein from unstimulated cells runs as two bands in a phos-tag gel (Figure S4), reflecting the phosphorylation state of unactivated MEK1 in mammalian cells.

**Figure 4:**
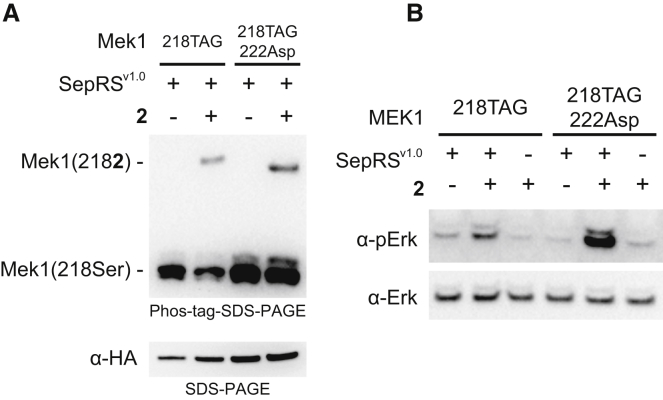
Activation of Mek by Incorporation of 2 (A) Phos-tag SDS-PAGE analysis is consistent with incorporation of **2** at position 218 in Mek1/2 (top). SDS-PAGE analysis (bottom) shows single band, confirming that the slower migration of upper band is due to chelation of phosphate group in the gel. (B) Expression of MEK1(218**2**) and MEK1(218**2**222D) results in phosphorylation of Erk in SepRS^v1.0^ dependent manner, as confirmed by phosphospecific antibody (top). The loading control was performed with standard Erk antibody (bottom). See also Figure S4.

We introduced plasmids expressing MEK1(218TAG) or MEK1(218TAG222Asp), along with an (ERK)-GFP fusion, SepRS^v1.0^, EF-1α-Sep, eRF1(E55D), tRNA^v1.0^_CUA_, and PSPH into HEK293/PSAT-KO cells. As expected, based on the GFP expression experiments, addition of **2** led to stably phosphorylated MEK variants, which were retarded in Phos-tag SDS-PAGE (Figure 4A) with respect to the bands characteristic of unactivated MEK.

Incorporation of **2** at position 218 of MEK led to phosphorylation of ERK-GFP (Figure 4B), consistent with reports that a serine to aspartic acid mutation at this position can stimulate some kinase activity (Huang et al., 1995). We observed a more dramatic increase in ERK-GFP phosphorylation by MEK(218**2**222D), consistent with maximal activity of MEK requiring both phosphorylations (Huang et al., 1995). Control experiments in which either SepRS^v1.0^ or **2** are omitted substantially decreased levels of ERK-GFP phosphorylation, further confirming that the activation we observe results from the genetically encoded incorporation of **2**.

## Discussion

We have demonstrated that the SepRS^v1.0^/tRNA^v1.0^_CUA_ pair is orthogonal in mammalian cells. We have developed an EF-1α mutant that facilitates the incorporation of phosphoserine, and combined this with engineered eRF1 and manipulations of phosphoserine biosynthesis to increase the efficiency of phosphoserine incorporation. By minimizing the intracellular pool of phosphoserine and feeding the cell a non-hydrolyzable analog of phosphoserine, we have been able to genetically encode a constitutive phosphorylation in mammalian cells. We anticipate that the first-generation system we have reported herein may be further optimized by directed evolution of the translational components we have developed herein in mammalian cells. In this regard, we note that the current PylRS/tRNA_CUA_ systems are orders of magnitude more efficient than the original systems (Schmied et al., 2014).

The demonstration that the SepRS^v1.0^/tRNA^v1.0^_CUA_ pair is orthogonal in mammalian cells and can be combined with other factors for the incorporation of pSer in mammalian cells, along with our recent demonstration that the SepRS^v1.0^/tRNA^v1.0^_CUA_ can be evolved in *E. coli* to recognize ncAAs that are not natural substrates of SepRS (Zhang et al., 2017) opens up many new opportunities. We note that it may be possible to evolve the SepRS^v1.0^/tRNA^v1.0^_CUA_ to incorporate ncAAs in *E. coli* and to use the evolved SepRS^v1.0^ variants to incorporate diverse ncAAs into proteins in mammalian cells.

We used our approach to activate MEK1 by co-translationally encoding a phosphorylated amino acid at genetically targeted sites *in vivo*. We anticipate that extensions of this approach may provide a route to deciphering the consequences of specific phosphorylations, both through synthetically manipulating cellular signaling pathways, as demonstrated here, and as a basis for capturing phospho-protein interaction partners in cells.

## Significance

**Protein phosphorylation is a key post-translational modification that has diverse roles in regulating the structure and function of proteins in eukaryotic cells. Natural phosphorylation is installed on certain amino acids within proteins by protein kinases and removed by protein phosphatases; most protein phosphorylation occurs at serine residues. Kinases commonly phosphorylate many substrates and several sites within a substrate; the ability to study the consequences of phosphorylation at specific sites in proteins would be enhanced by strategies to install serine phosphorylation, or analogs of serine phosphorylation into proteins by routes that are independent of protein kinases. Here we demonstrate that an aminoacyl-tRNA synthetase/tRNA**_**CUA**_ **pair, that we have previously developed in *E. coli* to site-specifically incorporate phosphoserine and its non-hydrolysable analog, can be used, in combination with other engineered translational machinery, to genetically encode the incorporation of phosphorylated amino acids into proteins in mammalian cells. While this first-generation approach may be improved by future modifications, we demonstrate its potential utility by installing a phosphorylated amino acid into the activation loop of a protein kinase, enabling its synthetic activation. The strategy we have reported, and its derivatives, may be used to synthetically control and understand kinase signaling, and the installation of non-hydrolyzable analogs may also enable biochemical and structural studies of phospho-protein complexes**.

## STAR★Methods

### Key Resources Table

**Table:** 

REAGENT or RESOURCE	SOURCE	IDENTIFIER
**Antibodies**		

Mouse monoclonal anti-GFP (clones 7.1 and 13.1)	Roche	Cat# 11814460001
Mouse monoclonal anti-FLAG (clone M2)	Sigma-Aldrich	Cat# F-1804
Mouse monoclonal anti-Myc (clone 9B11)	Cell Signalling	Cat# 2276
Mouse monoclonal anti -V5 (clone 2F11F7)	Invitrogen	Cat# 37-7500
Mouse monoclonal anti-actin (clone AC-40)	Sigma-Aldrich	Cat# A3853
Rat monoclonal anti-HA (clone 3F10)	Roche	Cat# 11 867 423 001
Mouse monoclonal anti-Erk1/2 (clone L34F12)	Cell Signalling	Cat# 4696
Anti-phosphoErk1/2 (clone D13.14.4E)	Cell Signalling	Cat# 4370
Rabbit polyclonal anti-PSPH	ThermoFisher	Cat# PA5-22003
Rabbit polyclonal anti-PSAT	Abcam	Cat# ab96136

**Bacterial and Virus Strains**		

E. coli BL21(DE3)	New England BioLabs	Cat# C2527
NEB Stable	New England BioLabs	Cat# C3040

**Chemicals, Peptides, and Recombinant Proteins**		

2 (DL-AP4)	Abcam	Cat# ab120001

**Critical Commercial Assays**		

AMP-Glo	Promega	Cat# V5011
CellTiter-Glo 2.0	Promega	Cat# G9241

**Experimental Models: Cell Lines**		

HEK293	ATCC	Cat# CRL-1573

**Oligonucleotides**		

Biotinylated northern blot probe: AAC CCA CGT AAG GCA ATT TTA GAG ACT GCT GCC TAA CCC CT	Integrated DNA Technologies	N/A
Fluorescent northern blot probe (quantitative): /5IDR800/GTG GGA TTT GAA CCC ACG TAA GGC AAT TTT	Integrated DNA Technologies	N/A
Guide RNA targeting PSPH exon 2 Top: CACCGCACACCTGACCCCCGGCATA Bottom: AAACTATGCCGGGGGTCAGGTGTGC	Integrated DNA Technologies	N/A
Guide RNA targeting PSAT exon 1 Top: CACCGCCAGGCAGGTGGTCAACTTT Bottom: AAACAAAGTTGACCACCTGCCTGGC	Integrated DNA Technologies	N/A

**Recombinant DNA**		

pET20b	Novagen	Cat# 69739-3
pcDNA3.4	ThermoFisher	Cat# A14697
(U6-PylT*)_4_/EF1α-PylRS	Schmied et al., 2014	N/A

**Software and Algorithms**		

Prism 7	GraphPad Software	https://www.graphpad.com/scientific-software/prism/

**Other**		

GFP-Trap MA beads	ChromoTek	Cat# gtma-20

### Contact for Reagent and Resource Sharing

Further information and requests for resources and reagents should be directed to and will be fulfilled by the Lead Contact, Jason Chin (chin@mrc-lmb.cam.ac.uk).

### Experimental Model and Subject Details

HEK293 cells (epithelial human embryonic kidney, female)(ATCC) were cultivated in DMEM (Gibco) supplemented with 10% fetal bovine serum (Gibco) in humifdified incubator at 37°C, 5% CO_2_. For passaging, cells were washed with phosphate buffered saline, detached using trypsin/EDTA-solution, resuspended in fresh growth medium and seeded into cell culture flasks. The cells were routinely tested for mycoplasma contamination.

### Method Details

#### Plasmid Generation

tRNA^v1.0^_CUA_ (B4 variant (Rogerson et al., 2015)) repeats containing the human U6 promoter, followed by the tRNA^v1.0^_CUA_ with no CCA tail, and a terminator were synthesized with unique homology arms on each side, allowing for cloning of four repeats by Gibson into a minimal backbone derived from pUC19. The resulting sequence of one repeat is as following:

##### Human U6 Promoter:: tRNA^v1.0^_CUA_:: Terminator

CCTAGTTGGGCAGGAAGAGGGCCTATTTCCCATGATTCCTTCATATTTGCATATACGATACAAGGCTGTTAGAGAGATAATTAGAATTAATTTGACTGTAAACACAAAGATATTAGTACAAAATACGTGACGTAGAAAGTAATAATTTCTTGGGTAGTTTGCAGTTTTAAAATTATGTTTTAAAATGGACTATCATATGCTTACCGTAACTTGAAAGTATTTCGATTTCTTGGCTTTATATATCTTGTGGAAAGGACGAAACACCGCCGGGGTAGTCTAGGGGTTAGGCAGCAGTCTCTAAAATTGCCTTACGTGGGTTCAAATCCCACCCCCGGCTGACAAGTGCGGTTTTT.

The structure of EF-1α was aligned with EF-Sep, and the engineered mutations were transferred over (see Figures S1A and S1B). The mutations (L77R, Q251N, D252G, V264S, N307W) were introduced via QuickChange site-directed mutagenesis and the resulting EF-1α-Sep was cloned into pcDNA3.4 via Gibson. SepRS^v1.0^ was cloned into pcDNA3.4 via Gibson from previously published plasmids (Rogerson et al., 2015).

Plasmids containing four copies of tRNA^v1.0^_CUA_, protein of interest (SepRS^v1.0^, EF-1α-Sep, or multiple cloning site (MCS)) under EF1A promoter were cloned by Gibson assembly from the above described plasmids and previously published plasmids (Schmied et al., 2014). The resulting constructs are designated pPB_FLAG-SepRS^v1.0^_4xU6-tRNA^v1.0^_CUA_, pPB_Myc-EF-1α-SepRS_4xU6-tRNA^v1.0^_CUA_ and pPB_MCS_4xU6-tRNA^v1.0^_CUA_. Fluorescence reporter constructs pPB_GFP(150TAG)_4xU6-tRNA^v1.0^_CUA_ (GFP refers to sfGFP variant (Pédelacq et al., 2006)) with C-terminal His_6_ tag) and pPB_mCherry-TAG-GFP_4xU6-tRNA^v1.0^_CUA_ were assembled from previously reported plasmids (Elsässer et al., 2016) via restriction cloning into pPB_MCS_4xU6-tRNA^v1.0^_CUA_. pPB_V5-eRF1(E55D)_4xU6-tRNA^v1.0^_CUA_ was similarly cloned via restriction cloning into pPB_MCS_4xU6-tRNA^v1.0^_CUA_. Control plasmids with no tRNA^v1.0^_CUA_ (pPB_MCS and pPB_mCherry-TAG-GFP) were cloned using Gibson assembly from the plasmids described above. pPB_FLAG-SepRS_Myc-EF1a_V5-eRF1(E55D)_4xU6-tRNA^v1.0^_CUA_ and pPB_FLAG-SepRS_Myc-EF1a_V5-eRF1(E55D)_V5-PSPH_4xU6-tRNA^v1.0^_CUA_ were enzymatically assembled via Gibson from plasmids described above with individual coding DNA sequences joined via cleavable P2A peptides. pPB_MEK1(218TAG)_4xU6-tRNA^v1.0^_CUA_ and pPB_MEK1(218TAG222Asp)_4xU6-tRNA^v1.0^_CUA_ were cloned by gibson assembly from previously reported plasmids (Tsai et al., 2015).

#### Phosphoserine Incorporation

HEK293 cells were seeded into poly-L-lysine coated 24 well plates (1.4 × 10^5^ cells per well). Upon reaching 70%–80% confluency cells were transfected with combinations of pPB_FLAG-SepRS^v1.0^_4xU6-tRNA^v1.0^_CUA_, pPB_Myc-EF-1α-Sep _4xU6-tRNA^v1.0^_CUA_, pPB_V5-eRF1(E55D)_4xU6-tRNA^v1.0^_CUA_ and pPB_mCherry-TAG-GFP_4xU6-tRNA^v1.0^_CUA_. For data in Figure 1, we transfected 125 ng of each plasmid per well, keeping the total amount of transfected DNA at 500 ng by addition of pPB_MCS_4xU6-tRNA^v1.0^_CUA_ or pPB_MCS. The DNA was transfected using 2 μL of 1 mg/ml solution of polyethylenimine MW 40,000 (PEI; Polysciences) per well. For subsequent experiments, the cells were transfected with combination of pPB_FLAG-SepRS_Myc-EF1a_V5-eRF1(E55D)_V5-PSPH_4xU6-tRNA^v1.0^_CUA_ or SepRS_Myc-EF1a_V5-eRF1(E55D)_V5-PSPH_4xU6-tRNA^v1.0^_CUA_ and pPB_GFP(150TAG)_4xU6-tRNA^v1.0^_CUA_ using 1000 ng of DNA/well. DNA and PEI were diluted in Opti-MEM and incubated for 15 minutes at room temperature. Cells were washed once with Opti-MEM before adding the transfection mix to the cells. The transfection medium was exchanged for fresh growth medium after 4 hours. The medium was exchanged again two days after the transfection. Cells were harvested and analyzed after 3 to 4 days of expression. Cell viability was measured using CellTiter-Glo 2.0 assay (Promega) according to the manufacturer’s instructions.

##### Flow Cytometry

The cells were washed with PBS, detached using trypsin/EDTA solution, resuspended in growth medium, pelleted and resuspended in PBS. The cells were analyzed using Becton Dickinson LSRII SORP (488 nm coherent sapphire laser for GFP excitation, 561 coherent compass laser for mCherry excitation). The data was analyzed in FlowJo software (FlowJo, LLC).

##### Western Blotting

For lysis HEK293 cells were washed with PBS and lysed in RIPA buffer containing HALT Protease and Phosphatase Inhibitor Cocktail (Thermo) and incubated at 4°C for 5 minutes. Samples were centrifuged for 10 minutes at 20000 rcf. Protein concentration of the supernatant was determined via BCA assay (ThermoFisher) according to manufacturer's instructions. The sample was transferred to SDS sample buffer (62.5 mM Tris-HCl (pH 7.4), 12.6% glycerol, 2% SDS, 100 mM DTT, 0.01% bromophenol blue) and boiled at 95°C for 5 minutes. Proteins were separated via SDS-PAGE using 4-12% Bis-Tris gels (Novex) and transferred to nitrocellulose membranes using the iBlot2 Dry Blotting System (ThermoFisher) according to manufacturer's instructions.

The membrane was blocked with 5% milk in tris-buffered saline with 0.1% Tween 20 (TBS-T) for 1 h and incubated with the primary antibody α-GFP (Roche (13.1 and 7.1)), α-FLAG (Sigma-Aldrich (F1804)), α-Myc (Cell Signaling (9B11)), α-V5 (Invitrogen (2F11F7)), α-actin (Sigma-Aldrich (A3853)), α-HA (Roche (3F10)), α-Erk (Cell Signalling (L34F12)), α-pErk (Cell Signalling (D13.14.4E)) at 4°C overnight. The membrane was washed several times with TBS-T and incubated with corresponding secondary antibody conjugated to HRP (Santa Cruz Biotechnology or Cell Signalling) for 1 h at room temperature. The blot was washed with TBS-T and developed using SuperSignal West Pico or Femto Chemiluminescent Substrate (ThermoFisher) according to manufacturer's instructions. Images were acquired using a ChemiDoc system (Bio-Rad).

##### Northern Blotting

RNA was isolated from HEK293 cells using the QIAzol Lysis Reagent (QIAGEN) according to manufacturers instructions. The samples were analyzed using the NorthernMax-Gly procedure (Thermo Fisher). Sep-tRNA was detected using a biotin labeled probe (AACCCACGTAAGGCAATTTTAGAGACTGCTGCCTAACC-CCT), which was hybridized at 37°C overnight in Ambion ULTRAhyb Ultrasensitive Hypridization Buffer (ThermoFisher). Blots were washed with low stringency wash buffer (2x saline-sodium citrate, 0.1% sodium dodecyl sulfate), incubated with horseradish peroxidase conjugated streptavidin and developed using enhanced chemiluminescence solution (both ThermoFisher).

#### Cell Line Generation

To knock out PSPH in HEK293 cells a guide RNA targeting exon 2 of PSPH was designed using to previously reported software (Hsu et al., 2013) and cloned into the pSpCas9(BB)-2A-GFP plasmid using a previously reported protocol (Ran et al., 2013) (top strand: CACCGCACACCTGACCCCCGGCATA; bottom strand: AAACTATGCCGGGGGTCAGGTGTGC). HEK293 cells were transfected with the plasmid using PEI and grown in DMEM containing 10% FBS and 1 mM serine for 3 days before they were sorted to single cells via fluorescence activated cell sorting based on GFP fluorescence. Subsequently, single cell colonies were screened for indel formation using the Guide-it mutation detection kit (Clontech laboratories) and a complete PSPH knock out was confirmed by western blot analysis using α-PSPH antibody (ThermoFisher). The PSAT knockout HEK293 cell line (HEK293/PSAT-KO) was created analogously using guide RNA targeting exon 1 of PSAT (top strand: CACCGCCAGGCAGGTGGTCAACTTT; bottom strand: AAACAAAGTTGACCACCTGCCTGGC). The primers used for genotyping via the Guide-it mutation detection kit were: CAGCCTGTTAATGTTATTTTCAAGC (PSPH exon 2 forward primer), ACGCTGTAGTAAGCCATGATTATAC (PSPH exon 2 reverse primer), AAACAGTAAACGCGAGGAGG (PSAT exon 1 forward primer), CTCATTCACACTATGTCCATTCATGC (PSAT exon 1 reverse primer).

#### In Vitro Experiments

##### SepRS Purification

The gene fragment coding for SepRS^v1.0^ was cloned into a pET-20b vector to give pET20b_SepRS. BL21(DE3) cells transformed with pET20b_SepRS were used to inoculate 2-litre 2xTY culture. At OD600 = 0.5, 0.1 mM IPTG was added and the culture was incubated at 24°C for 16hrs before being pelleted. The cell pellet was resuspended in lysis buffer (50 mM Tris-HCl, pH = 8.0, 300 mM NaCl, 20 mM imidazole, 1 mM beta-mercaptoethanol). After sonication, the lysates were heated to 70°C for 20 min and centrifuged at 20 000 g for 30 min. The supernatant was loaded onto a HisTrap column which was eluted by a linear gradient between 20 mM to 500 mM imidazole. The fractions with SepRS^v1.0^ were pooled and incubated with 3C protease at 4°C for 24hrs to remove the tag. Then, proteins were further loaded to HiTrap Q column and eluted with a gradient from 50 mM to 1 M NaCl. The fractions with SepRS^v1.0^ were pooled, concentrated, and snap frozen in liquid nitrogen and stored at -80°C.

##### tRNA Extraction and Purification

HEK293 cells were transfected as described above with plasmid containing 4xU6-tRNA^v1.0^_CUA_ cultured for 2 days and harvested by pipetting into ice cold PBS. 500 μl of cell pellet are resuspended in 6 ml of tRNA Buffer (50 mM NaOAc pH 5, 150 mM NaCl, 10 mM MgCl2, 0.1 mM EDTA) and aliquoted into 5x2 ml vial. The cells are centrifuged again (5 min, 500 rcf), the supernatant removed and 950 μl of fresh tRNA buffer are added. To lyse the cells, 50 μl of liquefied phenol (P9346, Sigma Aldrich) are added and the tubes put in mild head-over-tail shaking (10 rpm) for 15 min. The vials are then centrifuged for 25 min at 4°C at 21,000 rcf, then the supernatant containing the tRNAs is recovered and transferred to a new vial, where 1 volume of chloroform is added. After thorough mixing using a vortex, the emulsion is separated in a centrifuge (1 min, 21,000 rcf) and the acqueous phase recovered. To deacylate the purified tRNAs, 40 μl of NaOH 300 mM are added to 680 μl of aqueous phase. The reaction is incubated for 1 h at 42°C, then the solution is neutralised using 40 μl of NaOAc 3 M pH 5. The deacylated tRNAs are ethanol precipitated for 1 h at 10°C by adding 2.33 volumes of absolute ethanol to the recovered aqueous phase. After 30 min centrifugation at 21,000 rcf at 4°C the supernatant is removed and the precipitated RNAs resuspended in water.

##### *In Vitro* Aminoacylation Assay

The aminoacylation by SepRS^v1.0^ was measured with AMP-Glo assay (Promega). 25 nM recombinant SepRS^v1.0^ was mixed with 0, 3.3, 10, or 30 μg extracted tRNA in aminoacylation buffer (50 mM Tris pH = 7.4, 20 mM KCl, 2 mM DTT, 10 mM MgCl2) supplemented with 0.1 mM ATP, 1 mM phosphoserine, and 0.1 mg/ml BSA. Reactions were assembled to 25 μl and were incubated at 37°C for 2 hrs. The AMP generated by the reaction was then measured with AMP-Glo assay by following the instruction from the manufacturer.

#### Quantitative Northern Blot Analysis

500 ng of the RNAs purified from wild type cells or cell transfected with a plasmid containing the tRNA^v1.0^_CUA_ are run alongside linear dilutions of RNA standard on a 10% acrylamide gel under denaturing conditions (7 M urea, 1xTBE buffer) for 70 min at 200 V using the BioRad Mini-proean apparatus. The gel is then stained using SYBR Gold and imaged under blue light to visualise total RNA, then the RNA is transferred on positively charged nylon membrane using iBolt transfer system and crosslinked to the membrane under UV light. The membrane was pre-hypridised using the ULTRAhyb®-Oligo Buffer for 30 min at 37°C, then 2 μg of infrared fluorescent probe [5'-/5IDR800/GTG GGA TTT GAA CCC ACG TAA GGC AAT TTT -3', Integrated DNA Technologies] were added. The hybridisation was carried out at 37°C overnight. The excess probe was washed using 2xSSC buffer containing 0.1% SDS four times, then the membrane imaged using the Amersham Typhoon 5 (785 nm laser).

#### LC-MS Analysis of Intracellular Amino Acids

HEK293, HEK293/PSPH-KO and HEK293/PSAT-KO cells were cultured and transfected as described above. Cells were detached using trypsin and washed three times with PBS before analysis. Cells were counted using Countess II cell counter (ThermoFisher) prior to last pelleting of the cells. The frozen cell pellets were thawed at room temperature and resuspended in methanol-water (40:60) to 500 μl. The suspension was transferred to a 1.5-ml eppendorf tube. The cells were lysed by six freeze-thaw cycles (liquid nitrogen and room temperature). The resulting lysate was centrifuged at 21,000 g for 1 h at 4°C. The supernatant was pipetted into a 3K MWCO amicon centrifugal filter. The samples were centrifuged at 14,000 g for 30 min at 4°C. The flow-throw was analysed by LC-MS analysis. From the flow-through, 50 μl aliquots were pipetted into 250-μl glass inserts (Agilent). To prepare a calibration curve, 50-μl aliquots of the control sample were spiked with serine, pSer, or **2** to a final concentration of 5, 10, 20, 40, or 80 μM. For comparison, analogous calibration curves were also prepared in a methanol-water mixture (40:60).

An Agilent 1260 Infinity equipped with an Agilent 6130 Quadrupole LC-MS unit was used for analysis of all samples. From each sample, 10 μl was injected onto a Zorbax SB C18 column, 4.6 x 150 mm equipped with a guard column (Agilent). Each sample was eluted from the column using a mobile phase gradient from 0.5 % to 95 % acetonitrile containing 0.02 % formic acid. The mass spectrometer was set to selected ion monitoring (SIM) mode. For pSer, the ions measured were 186 M/z in the positive mode and 184 M/z in the negative mode. For Ser, the ions measured were 106 M/z in the positive mode and 104 M/z in the negative mode. Lastly, for **2**, the ions measured were 184 M/z in the positive mode and 182 M/z in the negative mode. The peaks for phosphoserine and serine overlapped with background peaks. Thus difference spectra were prepared for analysis by subtracting the chromatogram of the control with the chromatograms from each calibration standard. The resulting traces were integrated to derive a calibration curve. For the pSer lysate samples, the chromatograms from the HEK293 samples were subtracted from the chromatograms of the HEK293/PSPH-KO cell line. From the difference chromatograms, the unobscured peak for phosphoserine was integrated. A linear fit of the calibration curve from 10-80 μM was used to determine the concentration of serine, phosphoserine, and **2** in each of the lysates.

Using the following equation, the intracellular concentration was determined from the lysate concentration and the cell number measurements for each sample. [IC] = (lysate concentration × lysate volume) / (total number of cells × cell volume). The lysate volumes were 500 μl for all experiments. An approximate value was used for the cell volume, 2 pL. The intracellular concentration of phosphoserine in the control HEK cells was roughly estimated by extrapolation of the calibration curve and comparison with the 5 μM calibration point.

#### Purification of GFP from Mammalian Cells

HEK293 or HEK23/PSAT-KO cells were seeded into T-75 flasks (5.25 × 10^6^ per flask) 24 hours before transfection with 18.75 μg of DNA (75 μL PEI). The medium was changed after 4 hours to medium with or without 10 mM **2**, neutralized with NaOH. Cells were harvested, lysed and GFP was purified via GFP pull-down using GFP-Trap_MA beads (ChromoTek GmbH) according to manufacturer's instructions 3 days after transfection. Proteins were eluted by addition of SDS-sample buffer and boiling at 95°C for 10 minutes. Alternatively, proteins were eluted using 200 mM glycine buffer (pH 2), which was neutralized by addition of Tris HCl (pH 10.4). The proteins were separated via SDS-PAGE, stained using InstantBlue (Expedeon).

#### Phos-tag SDS-PAGE Electrophoresis

Cells were cultured and transfected as described above. Before harvesting, cells were washed twice with TBS and detached using pipette or cell scraper. The cells were lysed in RIPA buffer supplemented with HALT protease and phosphatase inhibitors (ThermoFischer). Protein concentration was measured using BCA assay (ThermoFischer) according to manufacturer’s instructions. 8% Phos-tag SDS-PAGE gels (Wako) were run according to manufacturer’s instructions (25 mA, 100-120 minutes) and the proteins were transferred onto PVDF membrane using iBlot2 system (ThermoFischer). The membrane was immunostained and developed as described above. GFP, GFP150pSer and GFP150(**2**) standards were expressed in *E. Coli* as described previously (Rogerson et al., 2015). Quantitative analysis was done using ImageJ.

#### LC-MS/MS Analysis of Amino Acid Incorporation

The tandem mass spectrometry was carried out as described previously (Rogerson et al., 2015). Briefly, bands of interest (1–2 mm) were cut from polyacrylamide gel. The gel slices were destained with 50% v/v acetonitrile and 50 mM ammonium bicarbonate, reduced with 10 mM DTT and alkylated with 55 mM iodoacetamide in 96-well plate. The proteins were digested overnight at 37°C using 6 ng/μl trypsin solution (Promega) and the resulting peptides were extracted with 2% v/v formic acid, 2% v/v acetonitrile. The peptides were analyzed by nanoscale capillary LC-MS/MS using Ultimate U3000 HPLC (ThermoScientific Dionex) under 300 nl/min flow. Before separation, the peptides were trapped on a C18 Acclaim PepMap100 3 μm, 75 μm × 150 mm nanoViper (ThermoScientfic Dionex) and subsequently eluted with acetonitrile gradient into a modified nanoflow ESI source with a hybrid dual pressure linear ion trap mass spectrometer (Orbitrap Velos, ThermoScientific). Analysis was carried out using a resolution of 30,000 for the full MS spectrum followed by ten MS/MS spectra in the linear ion trap using 35 as threshold energy of for collision-induced dissociation.

For the targeted analysis of the GFP tryptic peptide LEYNFNSH[X]VYITADK, where X=glutamine, serine, phosphoserine or **2**, the theoretical masses were determined to 4 significant figures for all relevant charge states and the raw data searched with a m/z range of ±0.15 Da. The resulting extracted ion chromatograms were integrated and the area-under-the-curve (AUC) was used for relative quantitation.

The collected LC-MS/MS data were searched using the Mascot search engine (Matrix Science) against database containing Swiss-Prot and the GFP construct with all amino acids and post-translational modifications allowed at position 150. Precursor tolerance of 5 p.p.m. and a fragment ion mass tolerance of 0.8 Da were used for the search, allowing two missed enzyme cleavages and variable modifications for oxidized methionine, carbamidomethyl cysteine, pyroglutamic acid and phosphorylated serine, threonine and tyrosine. MS/MS data were subsequently validated using the Scaffold program (Proteome Software Inc.) and interrogated manually.

#### MEK1 Expression

HEK293/PSAT-KO cells were seeded and transfected as described above with combination of pPB_FLAG-SepRS_Myc-EF1a_V5-eRF1(E55D)_V5-PSPH_4xU6-tRNA^v1.0^_CUA_, Erk-GFP reporter and pPB_MEK1(218TAG)_4xU6-tRNA^v1.0^_CUA_ or pPB_MEK1(218TAG222Asp)_4xU6-tRNA^v1.0^_CUA_. The cells were incubated in DMEM supplemented with 10% FBS and 10 mM **2**, lysed and analysed by phos-tag-SDS-PAGE or SDS-PAGE and western blotting, as described above.

### Quantification and Statistical Analysis

Quantification of western blots was carried out using ImageJ. Details of all statistical datasets are included in the corresponding figure legends.

## Supplemental Information

Document S1. Figures S1–S4

Document S2. Article plus Supplemental Information
