# Supplementary material for: Genetically Encoded Protein Phosphorylation in Mammalian Cells
Source: Cell Chem Biol. 2018 Sep 20;25(9):1067–1074.e5. doi: 10.1016/j.chembiol.2018.05.013 (PMC6162345; doi:10.1016/j.chembiol.2018.05.013)
Supplement: Document S1. Figures S1–S4 [file mmc1.pdf]

Cell Chemical Biology, Volume *25*

Supplemental Information

Genetically Encoded Protein Phosphorylation in Mammalian Cells

Václav Beránek, Christopher D. Reinkemeier, Michael S. Zhang, Alexandria D. Liang, Gene Kym, and Jason W. Chin

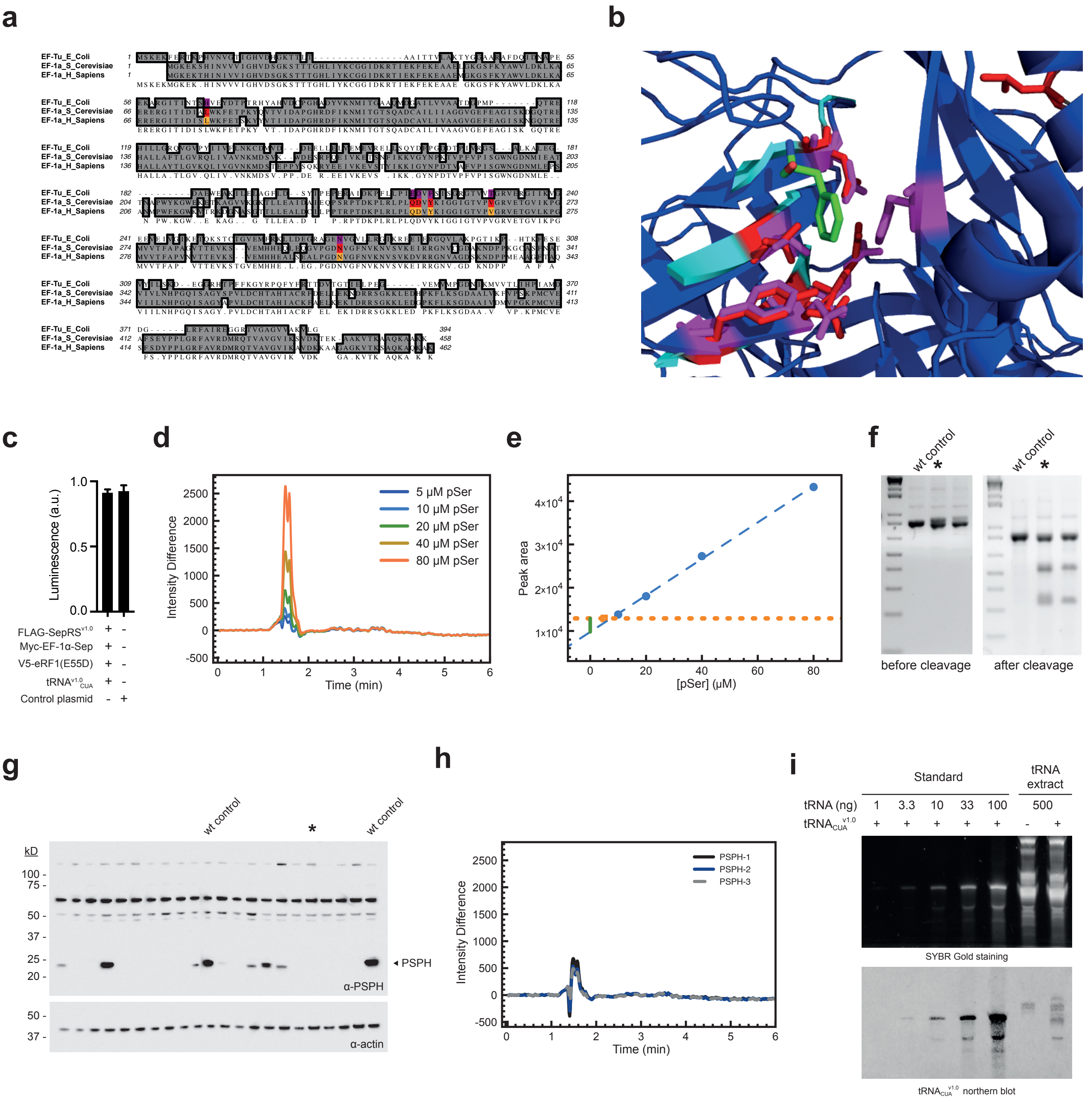

Figure S1. The SepRS$^{v1.0}$/tRNA$^{v1.0}_{CUA}$ pair enables pSer incorporation in mammalian cells. Related to Figure 1. (**a**) Clustal alignment of protein sequences of EF-Tu (*E. coli*) and EF-1α (*S. cerevisiae* and *H. sapiens*). Residues mutated to create EF-Sep (Park et al., 2011) (H67R, E216N, D217G, F219Y, T229S, N274W) are higlighted in magenta, corresponding residues in EF-1α are highlighted in red (*S. cerevisiae*) and orange (*H. sapiens*). (**b**) Aligned structures of *E. coli* EF-Tu in complex with phenylalanyl-tRNA (PDB:1ob2; EF-Tu in blue, Phe in green, tRNA not shown) and *S. cerevisiae* EF-1α (PDB:1f60; cyan, only structure surrounding the critical residues shown). Mutated residues are highlighted in magenta (*E. coli*) and red (*S. cerevisiae*). (**c**) CellTiter-Glo assay confirms that cell viability is not compromised by transfection of the genetic code expansion components when compared to transfection of a control plasmid. (**d**) The LC-MS chromatograms obtained from subtracting the unspiked HEK293 lysates from each of the pSer-spiked samples are graphed as a function of time. (**e**) The peak area of each pSer-spiked sample plotted as a function of the spiked pSer concentration. The original chromatograms were used for analysis rather than the difference data. The dashed, blue line represents a linear fit from 10-80 μM pSer. The dotted orange line represents the peak area for the 5 μM pSer standard. The green line represents the difference between the peak area for 5 μM pSer and the projected peak area for a sample with no pSer spiked in, corresponding to approx. 100 μM pSer. (**f**) Characterization of HEK293/PSPH-KO. Gel electrophoresis of PCR amplicons of PSPH exon 2 from HEK293/PSPH-KO clonal cell lines before (left) and after reannealing and treatment with Guide-it resolvase (right). Star denotes the final clone used in this study. (**g**) Confirmation of PSPH knockout using western blot. Star denotes the final clone used in this study. (**h**) The LC-MS chromatograms obtained from subtracting the unspiked HEK293 lysates from each of the HEK293/PSPH-KO lysate samples. Peak areas correspond to increase in pSer concentration of 400 ± 60 μM (SD) over HEK293. (**h**) Northern blot confirms presence of tRNA$^{v1.0}_{CUA}$ in mammalian tRNA extract. SYBR Gold staining of RNA after denaturing PAGE (top panel). Lanes 1 to 5 contain fixed amounts of a chemically synthesised RNA oligo with the same sequence of sepT in the range from 1 to 100 ng. The last two lanes contain a total of 500 ng of RNA extract (measured by UV absorbance) from wild type HEK293 cells, or from cells transfected with plasmid containing 4x tRNA$^{v1.0}_{CUA}$ gene under u6 promoter. Northern blot using a fluorescently labelled probe specific for tRNA$^{v1.0}_{CUA}$ (bottom panel). No specific signal can be detected from the RNA extract of wild type cells. The signal detectable from the tRNA$^{v1.0}_{CUA}$ expressing cells is slightly greater than the one measured from the standard containing 10 ng of RNA, but lower than the one from the standard containing 33 ng of RNA.

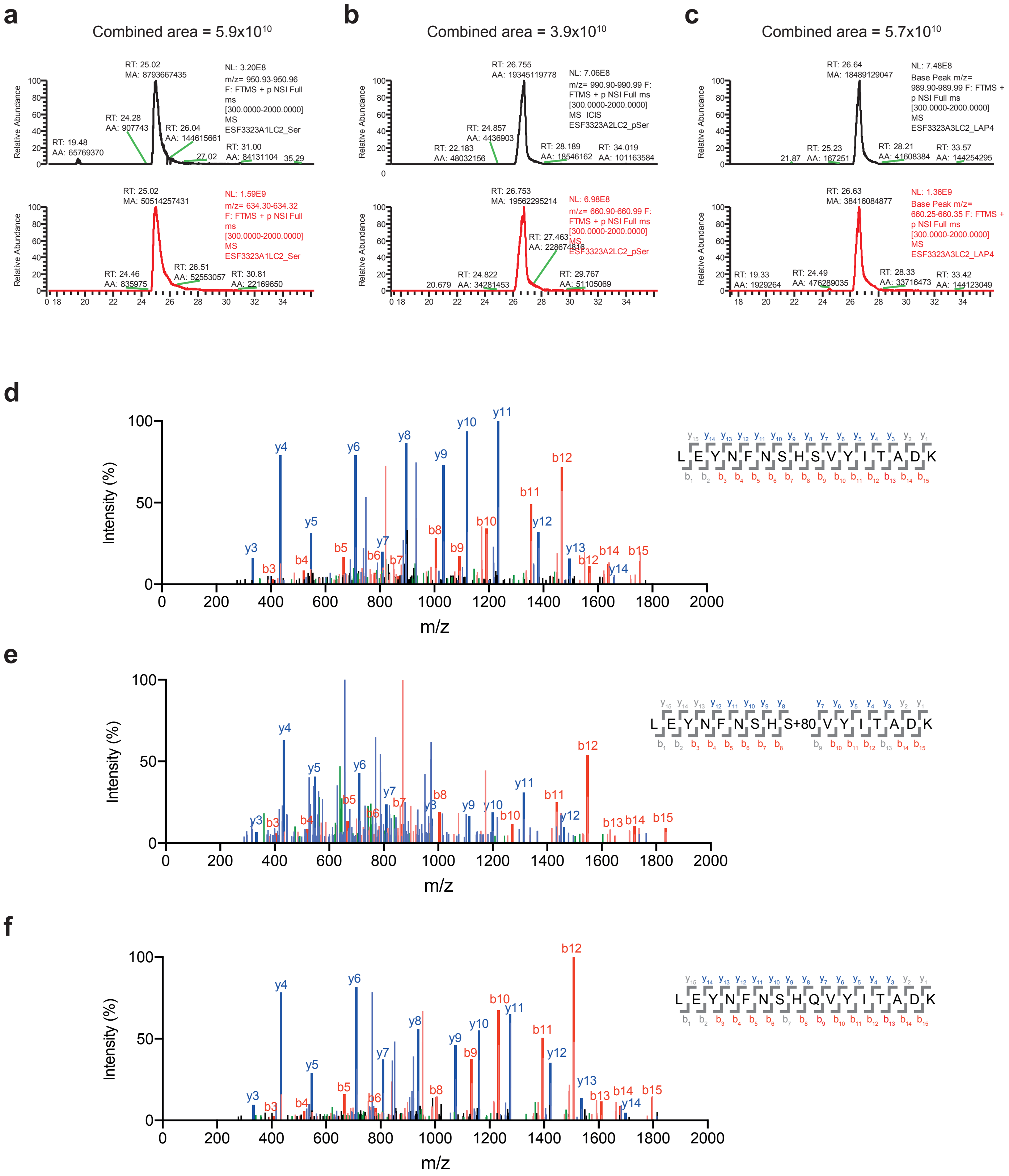

Figure S2. LC-MS quantification and LC-MS/MS identification of serine, phosphoserine and 2 incorporation using E. coli standards. Related to Figure 2. Extracted ion chromatograms of 2+ (black line) and 3+ (red line) ions of the tryptic peptides LEYNFNSH[X]VYITADK where (**a**) [X] = serine, (**b**) [X] = phosphoserine and (**c**) [X] = **2** when the instrument was injected with 1:1:1 mixture of GFP150Ser, GFP150pSer and GFP150**2**. Combined areas under the curves for 2+ and 3+ ions (shown in figures) demonstrate that the ionization of the three peptides is comparable. MS/MS spectrum confirms incorporation of serine (**d**), phosphoserine (**e**) and glutamine (**f**) at position 150 in sfGFP150TAG extracted from mammalian cells. We detected no other amino acids at the position 150.

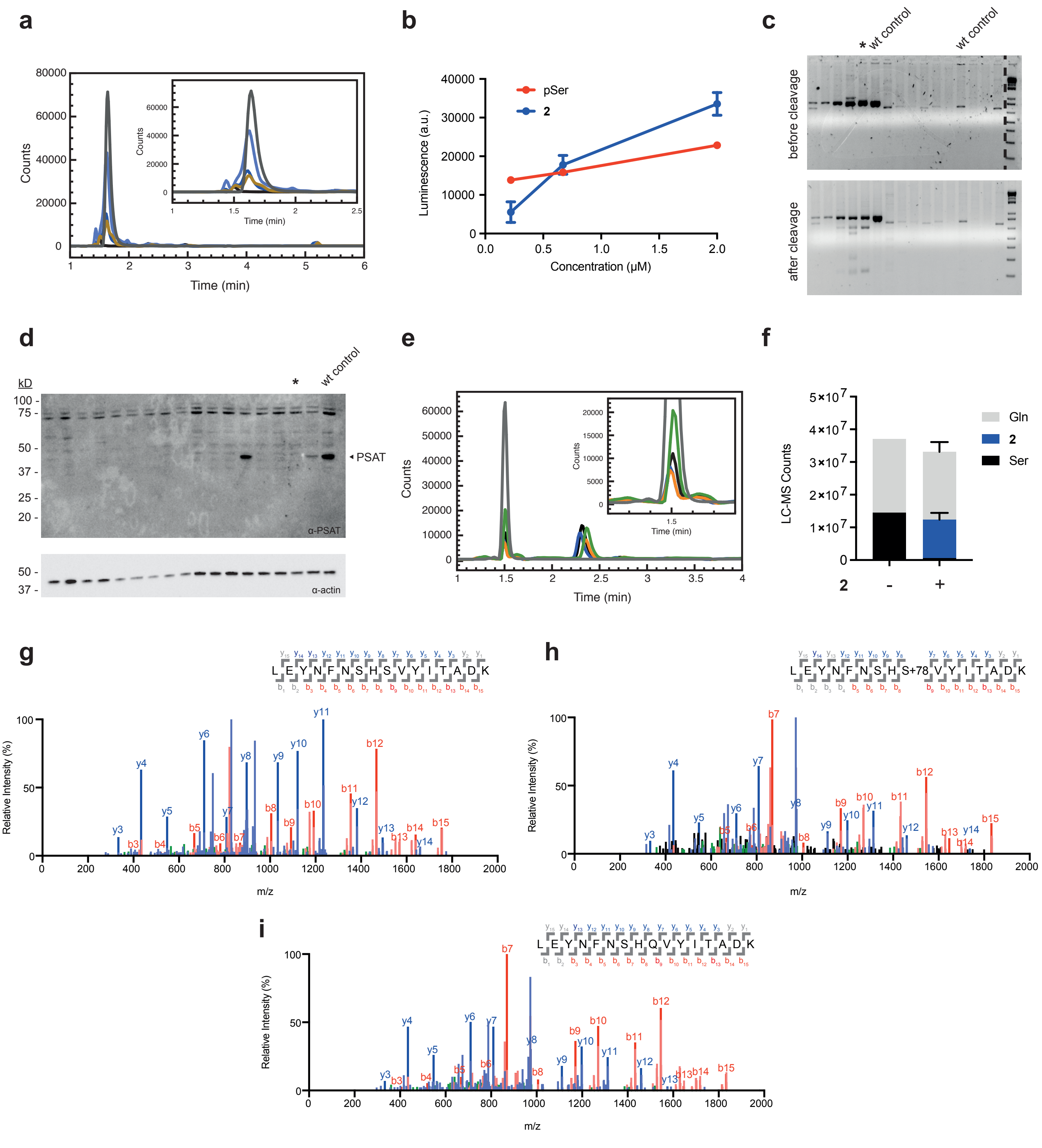

Figure S3. Encoding non-hydrolyzable phosphonate (2) in genetically engineered mammalian cells. Related to Figure 3. (**a**) Phosphonate analogue is taken up by HEK293 cells from culture media. The LC-MS chromatograms of lysates from wtHEK293 cultured in standard growth media (black), wtHEK293 supplemented with 2 mM **2** in growth media (dark blue), wtHEK293 supplemented with 20 mM **2** in growth media (light blue), wtHEK293 with 500 µM **2** added to the lysate (final concentration) (gold) and aqueous standard of **2** (grey). Peak areas correspond to intracellular concentration of 3.64 ± 0.39 mM and 12.47 ± 0.80 mM intracellular **2** for cells cultured in media supplemented with 2 mM and 20 mM **2**, respecitvely. All values represent mean ± SD from three biological replicates. The insert is magnified section of the chromatograms. (**b**) **2** is a substrate for $SepRS^{DTR}$ in vitro. Recombinant $SepRS^{DTR}$ was incubated with total tRNA extracted from cells transfected with $tRNA_{CUA}^{DTR}$ and the reactions were supplemented with phosphoserine or **2** in varying concentrations. The aminoacylation in each reaction was determined by measuring the AMP production with an AMP-Glo assay. The luminescence of reactions was normalised by subtracting the luminescence of reaction with no amino acid. Data represent mean ± SEM for three replicates. (**c**) Characterization of HEK293/PSPH-KO and HEK293/PSAT-KO cell lines. Gel electrophoresis of PCR amplicons of PSAT exon 1 from HEK293/PSAT-KO clonal cell lines before (top) and after reannealing and treatment with Guide-it resolvase (bottom). Star denotes the clone used in this study. Dashed line indicates unrelated lanes that were removed for clarity. Dashed line indicates unrelated lanes that were removed for clarity. (**d**) Confirmation of PSAT knockout using western blot. Star denotes the clone used in this study. (**d**) Knockout of PSAT and overexpression of PSPH only slightly decreases the intracellular Serine concentration. (**e**) The LC-MS chromatograms of lysates from wtHEK293 (black), HEK293/PSAT-KO (dark blue), HEK293/PSAT-KO with overexpressed PSPH (orange), wtHEK293 with spiked 50 µM Serine (final concentration) (green) and aqueous standard for Serine (grey). Peak areas correspond to intracellular Serine concentration of 362 ± 52 µM, 201 ± 22 µM, 215 ± 14 µM for wtHEK293, HEK293/PSAT-KO and HEK293/PSAT-KO with PSPH overexpression, respecitvely. All values represent mean ± SD from three biological replicates. The insert is magnified section of the chromatograms. (**f**) **I**dentification of amino acid incorporated in response to amber codon in sfGFP150TAG. LC-MS counts of tryptic peptides containing serine, glutamine or **2** at position 150 in sfGFP (no other amino acids were detected). Data are represented as mean ± SEM, where relevant. MS/MS spectrum confirms incorporation of serine (**g**), **2** (**h**) and glutamine (**i**) at position 150. No other amino acids were detected.

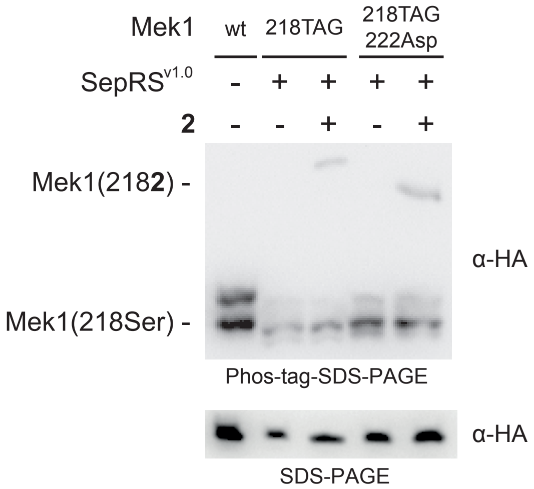

Figure S4. Activation of Mek by incorporation of 2. Related to Figure 4. Phos-tag SDS PAGE and SDS PAGE analysis of wtMek1/2, Mek1/2(218TAG) and Mek1/2(218TAG222Asp). wtMEK1/2 migrates in two bands on phos-tag SDS PAGE possibly due to endogenous phosphorylation. MEK1/2 was detected using C-terminal HA epitope tag.
